# Combination therapy for cancer with oncolytic virus and checkpoint inhibitor: A mathematical model

**DOI:** 10.1371/journal.pone.0192449

**Published:** 2018-02-08

**Authors:** Avner Friedman, Xiulan Lai

**Affiliations:** 1 Mathematical Bioscience Institute & Department of Mathematics, Ohio State University, Columbus, OH, United States of America; 2 Institute for Mathematical Sciences, Renmin University of China, Beijing, P. R. China; European Institute of Oncology, ITALY

## Abstract

Oncolytic virus (OV) is a replication competent virus that selectively invades cancer cells; as these cells die under the viral burden, the released virus particles proceed to infect other cancer cells. Oncolytic viruses are designed to also be able to stimulate the anticancer immune response. Thus, one may represent an OV by two parameters: its replication potential and its immunogenicity. In this paper we consider a combination therapy with OV and a checkpoint inhibitor, anti-PD-1. We evaluate the efficacy of the combination therapy in terms of the tumor volume at some later time, for example, 6 months from initial treatment. Since T cells kill not only virus-free cancer cells but also virus-infected cancer cells, the following question arises: Does increasing the amount of the checkpoint inhibitor always improve the efficacy? We address this question, by a mathematical model consisting of a system of partial differential equations. We use the model to construct, by simulations, an efficacy map in terms of the doses of the checkpoint inhibitor and the OV injection. We show that there are regions in the map where an increase in the checkpoint inhibitor actually decreases the efficacy of the treatment. We also construct efficacy maps with checkpoint inhibitor vs. the replication potential of the virus that show the same antagonism, namely, an increase in the checkpoint inhibitor may actually decrease the efficacy. These results have implications for clinical trials.

## Introduction

PD-1 is an immunoinhibitory receptor predominantly expressed on activated T cells [1, 2]. Its ligand PD-L1 is upregulated on the same activated T cells, and in some human cancer cells [2, 3]. The complex PD-1-PD-L1 is known to inhibit T cell function [1]. Immune checkpoints are regulatory pathways in the immune system that inhibit its active response against specific targets. In the case of cancer, the complex PD-1-PD-L1 functions as an immune checkpoint for anti-tumor T cells. CTLA-4 is another immunoinhibitory receptor expressed on activated T cells; when it combines with its ligand B7 on dendritic cells, the complex CTLA-4-B7 acts as a checkpoint inhibitor for anti-tumor T cells [4, 5]. There has been much progress in recent years in developing checkpoint inhibitors, primarily anti-PD-1 and anti-PD-L1 [6], and anti-CTLA-4 [7, 8].

Oncolytic virus (OV) is a genetically engineered virus that can selectively invade into and replicate within cancer cells while not harming normal healthy cells. OV therapy has been explored as an approach to combat cancer, and clinical trials were carried out on different types of cancer [9–12]. However, therapeutic efficacy remains a challenge [13, 14]. One of the factors that limits OV therapy is the antigenicity of the infected cells; the macrophages of the innate immune system recognize these cells and destroy them together with the virus particles inside them. For this reason, experimental studies considered combination of OV therapy with immune suppressive drugs [15–18].

In another direction, some studies consider OV with viruses designed to both replicate within cancer cells and stimulate cytotoxic T cells; such viruses include vesicular stomatitis virus [19, 20], Newcastle Disease Virus [21], vaccinia [22, 23], measle virus [24], and others [25, 26]. Advances in the design of various oncolytic viruses are reported in [27, 28]. The underlying assumption in these studies is that the virus will survive long enough, under the pressure of the innate immune attack, to activate a sufficiently large number of cytotoxic T cells that will eradicate or significantly reduce the cancer. To make this approach more effective, it was suggested to combine the OV drug with checkpoint inhibitors. Several mouse experiments, with different types of cancer cells, reported that both CTLA-4 and PD-L1 checkpoints blockade enhanced the OV therapy [29–33]. There are also several clinical trials with OV and checkpoint inhibitors [34–37].

In previous work the authors considered combination therapies with checkpoint inhibitor and, as a second agent, tumor vaccine [38] or BRAF inhibitor [39]. In the present paper the second agent is oncolytic virus. This poses a dilemma, since T cells kill not only virus-free cancer cells but also virus-infected cancer cells (thus reducing the anti-cancer effect of the virus), while checkpoint inhibitors enhance the T cells activities. Thus, it is natural to ask whether increasing the amount of the checkpoint inhibitor does always result in a decrease in tumor volume. We develop a mathematical model to address this question. We denote by *γ*_*V*_ the dose amount of the injected OV and by *γ*_*A*_ the dose amount of the checkpoint inhibitor, and define the efficacy of the treatment by (*γ*_*V*_, *γ*_*A*_) in terms of the tumor volume at some arbitrary time, for example, 24 weeks from the beginning of the treatment. We use the mathematical model to develop an efficacy map, and we find that there are regions in (*γ*_*V*_, *γ*_*A*_) plane where an increase in *γ*_*A*_ results in actual decrease in the efficacy. We denote by $\lambda_{V_{i}}$ the replication rate of viruses within infected cancer cells. We then construct efficacy maps for ($\lambda_{V_{i}}$, *γ*_*A*_) and find regions where an increase in *γ*_*A*_ again results in decreased efficacy. In such regions, the indiscriminent killing of infected and uninfected cancer cells has pro-cancer effect. These have implications for clinical trials.

**Fig 1:**
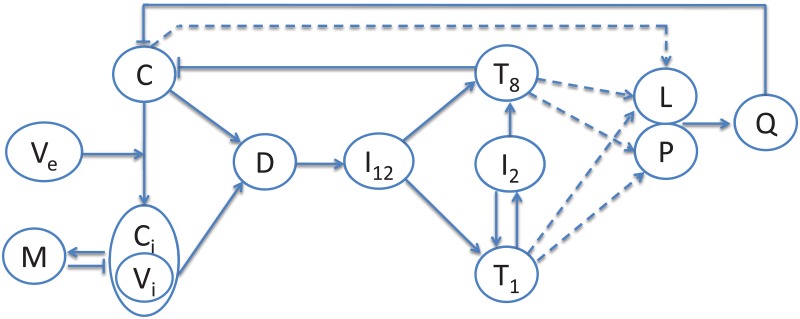
Interaction of tumor cells with virus and immune cells. Sharp arrows indicate proliferation/activation, blocked arrows indicate killing/blocking, and dashed lines indicate proteins on T cells. *C*: uninfected cancer cells, *C*_*i*_: infected cancer cells, *V*_*e*_: extracellular virus, *V*_*i*_: intracellular virus, *D*: dendritic cells, *T*_1_: CD4^+^ Th1 cells, *T*_8_: CD8^+^ T cells, *I*_2_: IL-2, *I*_12_: IL-12, *P*: PD-1, *L*: PD-L1, *Q*: PD-1-PD-L1 complex.

The mathematical model includes CD4^+^ Th1 cells and CD8^+^ T cells, macrophages, and dendritic cells. Dendritic cells are activated indirectly by the virus and by necrotic cancer cells, while macrophages are activated by virus-infected cancer cells. Macrophages engulf and destroy infected cancer cells, but they also kill, at a lesser rate, uninfected cancer cells. When a cancer cell is infected by an extracellular virus, the extracellular virus becomes an intracellular virus within the infected cell. Intracellular viruses multiply within the cancer cells and cause them to lyse, thereby releasing all their viruses to the extracellular environment. T cells are activated by IL-12 produced by dendritic cells, and they also proliferate by IL-2 produced by Th1 cells. Fig 1 shows the network of interactions among the cells, with PD-1 and PD-L1 on T cells and PD-L1 also on tumor cells.

We assume that the treatment with combination therapy extends over a period of 16 weeks, and we evaluate the results of the treatment at the end of 24 weeks. We can use the model to compute the tumor volume at the end of 24 weeks for each pair of parameters of ($\lambda_{V_{i}}$, λ_*DV*_) and doses (*γ*_*A*_, *γ*_*V*_).

The mathematical model is represented by a system of partial differential equations based on Fig 1.

## Mathematical model

The mathematical model is based on the diagram in Fig 1. The list of variables is given in Table 1, where the density of cells and concentration of cytokines are all in unit of g/cm^3^. The time unit is 1 day.

**Table 1:** List of variables (in units of g/cm^3^).

Notation	Description	Notation	Description
*C*	density of cancer cells	*T*_8_	density of activated CD8^+^ T cells
*C*_*i*_	density of infected cancer cells	*I*_12_	IL-12 concentration
*V*	density of extracellular virus	*I*_2_	IL-2 concentration
*V*_*i*_	density of intracellular virus	*P*	PD-1 concentration
*M*	density of macrophages	*L*	PD-L1 concentration
*D*	density of dentritic cells	*Q*	PD-1-PD-L1 concentration
*T*_1_	density of activated CD4^+^ T cells	*A*	anti-PD-L1 concentration

We assume that the total density of cells within the tumor remains constant in space and time, so that

$$\begin{array}{c} C+C_{i}+M+D+T_{1}+T_{8}=\text{constant}=\theta . \end{array}$$ (1)

Since cancer cells proliferate while T cells and macrophages enter the tumor, the assumption (1) implies that there is an internal pressure among cells, and this gives rise to a velocity **u** of cells.

**Equation for uninfected cancer cells (*C*)**. We assume a logistic growth for cancer cells, and that cancer cells are killed primarily by CD8^+^ T cells at a rate *η*_8_ *T*_8_ *C* where *η*_8_ is a constant. Cancer cells become infected by *V*_*e*_ at a rate proportional to *CV*_*e*_. Therefore *C* satisfies the following equation:

$$\begin{array}{c} \begin{array}{cc} \frac{\partial C}{\partial t}+\nabla \cdot \left(\text{u}C\right)-\delta_{C}\nabla^{2}C= & \underset{\text{growth}}{\underset{︸}{\lambda_{C}C\left(1-\frac{C}{C_{M}}\right)}}-\underset{\text{infection}\;\text{by}\;V_{\text{e}}}{\underset{︸}{\beta_{C}CV_{e}}}\\ & -\underset{\text{killed}\;\text{by}\;\text{CD}8^{+}\;\text{T}\;\text{cells}}{\underset{︸}{\eta_{8}T_{8}C}}-\underset{\text{death}}{\underset{︸}{d_{C}C}}, \end{array} \end{array}$$ (2)

where *δ*_*C*_ is the dispersion coefficient and *d*_*C*_ is the death rate by apoptosis.

**Equation for infected cancer cells (*C*_*i*_)**. We assume that CD8^+^ T cells kill infected cancer cells at a rate $\eta_{8C_{i}}T_{8}C_{i}$, where $\eta_{8C_{i}}$ is a constant larger than *η*_8_. We also assume that macrophages kill infected cancer cells by phagocytosis [40] at a rate proportional to *C*_*i*_ *M*. The death rate of infected cancer cells is larger than the death rate of uninfected cancer cells by a factor $\mu_{V_{i}}$ *V_i_* which represents the effect of viral burden. We take the rate by which cancer cells become infected to be *β*_*C*_ *CV*_*e*_. We finally assume that the density of the *V*_*i*_ cells is proportional to the density of the *C*_*i*_ cells within which the *V*_*i*_ reside, so that they have the same dispersion coefficient. Hence the equation for infected cancer cells is given by

$$\begin{array}{c} \begin{array}{cc} \frac{\partial C_{i}}{\partial t}+\nabla \cdot \left(uC_{i}\right)-\delta_{C_{i}}\nabla^{2}C_{i}= & \beta_{C}CV_{e}-\underset{\text{death}}{\underset{︸}{d_{C}\left(1+\mu_{V_{i}}V_{i}\right)C_{i}}}-\underset{\text{killed}\;\text{by}\;M}{\underset{︸}{\mu_{C_{i}M}C_{i}M}}\\ & -\underset{\text{killed}\;\text{by}\;\text{CD}8^{+}\;\text{T}\;\text{cells}}{\underset{︸}{\eta_{8C_{i}}T_{8}C_{i}}}. \end{array} \end{array}$$ (3)

**Equation for extracellular virus (*V*_*e*_)**. We assume that virus at amount *γ*_*V*_ is injected into the tumor at successive days *t*_1_, *t*_2_, …, *t*_*n*_. Thus at each day *t*_*j*_ we have to increase *V*_*e*_ by an amount *γ*_*V*_, that is, *V*_*e*_(*t*_*j*_ + 0) − *V*_*e*_(*t*_*j*_ − 0) = *γ*_*V*_. This increase can be written in the form

$$\frac{dV_{e}}{dt}|{}_{t=t_{j}}=\gamma_{V}\delta \left(t-t_{j}\right)$$

where *δ*(*s*) is the Dirac measure. We assume that when an infected cell dies the intracellular viral particles are released into the tumor microenvironment; however, when an infected cell is killed by macrophages or *T*_8_ cells, the virus particles inside it are cleared out. Extracellular virus are endocytosed by macrophages, and the rate of their depletion is proportional to *MV*_*e*_. Hence, the equation for *V*_*e*_ takes the following form:

$$\begin{array}{c} \begin{array}{cc} \frac{\partial V_{e}}{\partial t}-\delta_{V_{e}}\nabla^{2}V_{e}= & \sum_{j=1}^{m}\gamma_{V}\delta \left(t-t_{j}\right)+\underset{\text{released}\;\text{by}\;C_{i}\;\text{death}}{\underset{︸}{Nd_{C}\left(1+\mu_{V_{i}}V_{i}\right)V_{i}}}\\ & -\underset{{\text{V}}_{\text{e}}\to {\text{V}}_{\text{i}}}{\underset{︸}{\beta_{V}CV_{e}}}-\underset{\text{endocytosed}\;\text{by}\;M}{\underset{︸}{\mu_{V_{e}M}MV_{e}}}, \end{array} \end{array}$$ (4)

where *N* is the average number of viral particles released at death of an infected cancer cell. Note that the coefficient *β*_*V*_ is related to the coefficient *β*_*C*_ in Eq (2) by the equation *β*_*V*_ = *β*_*C*_ *m*_*VC*_, where *m*_*VC*_ is the ratio of the mass of one virus to one cancer cell.

**Equation for intercellular virus (*V*_*i*_)**. Viruses multiply in a cancer cell by exploiting the DNA of the cell as a ‘resource’. We represent the proliferation of the viruses in the cell by $\lambda_{V_{i}}$ *C_i_*. The equation for *V*_*i*_ is the following:

$$\begin{array}{c} \begin{array}{cc} \frac{\partial V_{i}}{\partial t}+\nabla \cdot \left(uV_{i}\right)-\delta_{C_{i}}\nabla^{2}V_{i}= & \underset{{\text{V}}_{\text{e}}\;\to \;{\text{V}}_{\text{i}}}{\underset{︸}{\beta_{V}CV_{e}}}+\underset{\text{growth}\;\text{of}\;V_{i}\;\text{in}\;C_{i}}{\underset{︸}{\lambda_{V_{i}}C_{i}}}-\underset{\text{released}\;\text{through}\;\text{death}\;\text{of}\;C_{i}}{\underset{︸}{Nd_{C}\left(1+\mu_{V_{i}}V_{i}\right)V_{i}}}\;\\ & -\underset{\text{killed}\;\text{by}\;M}{\underset{︸}{\mu_{C_{i}M}V_{i}M}}-\underset{\text{killed}\;\text{by}\;\text{CD}8^{+}\text{cells}}{\underset{︸}{\eta_{8C_{i}}T_{8}V_{i}}}. \end{array} \end{array}$$ (5)

The last two terms represent a loss of *V*_*i*_ due to death of their host *C*_*i*_ by the macrophages and CD8^+^ T cells. Note that *V*_*i*_ moves with the same velocity $\vec{u}$ as *C*_*i*_.

**Equation for macrophages (*M*)**. The growth rate of the proinflammatory macrophages is promoted by infected cancer cells and is represented by a term $\lambda_{MC_{i}}MC_{i}$. Hence *M* satisfies the following equation:

$$\begin{array}{c} \begin{array}{cc} \frac{\partial M}{\partial t}+\nabla \cdot \left(uM\right)-\delta_{M}\nabla^{2}M= & \underset{\text{Source}}{\underset{︸}{\lambda_{M}}}+\underset{\text{growth}}{\underset{︸}{\lambda_{MC_{i}}MC_{i}}}-\underset{\text{death}}{\underset{︸}{d_{M}M}}, \end{array} \end{array}$$ (6)

where λ_*M*_ is a source of macrophages prior to the treatment with OV.

**Equation for dentritic cells (*D*)**. Oncolytic virus is often armed to elicit adaptive immune response [19, 24]. In particular, we assume that inactive dendritic cells with density *D*_0_ are activated by intracellular armed viruses at a rate proportional to *D*_0_*V*_*i*_. Dendritic cells are also activated by HMGB-1 [41, 42], which is produced by necrotic cancer cells (NCs) [43]. We assume that the concentration of HMGB-1 is proportional to the density of NCs and that the density of NCs is proportional to the density of cancer cells. Hence, the activation rate of inactive dendritic cells is proportional to $D_{0}\frac{C}{K_{C}+C}$, where the Michaelis-Menten law is used to account for the limited receptor recycling time which occurs in the process of DC activation. The dynamics of DCs is given by

$$\begin{array}{c} \begin{array}{cc} \frac{\partial D}{\partial t}+\underset{\text{velocity}}{\underset{︸}{\nabla \cdot \left(uD\right)}}-\underset{\text{difusion}}{\underset{︸}{\delta_{D}\nabla^{2}D}}= & \underset{\text{activation}\;\text{by}\;\text{intracellular}\;\text{virus}}{\underset{︸}{\lambda_{DV}D_{0}V_{i}}}\;\\ & +\underset{\text{activation}\;\text{by}\;\text{HMGB}-1}{\underset{︸}{\lambda_{DC}D_{0}\frac{C}{K_{C}+C}}}\;-\underset{\text{death}}{\underset{︸}{d_{D}D}}, \end{array} \end{array}$$ (7)

**Equation for CD4^+^ T cells (*T*_1_)**. Naive CD4^+^ T cells are activated by IL-12 while in direct contact with dendritic cells. IL-2 induces proliferation of activated *T*_1_ cells [44, 45]. Both processes are inhibited by the complex PD-1-PD-L1 (*Q*) [46], by a factor $\frac{1}{1+Q/K_{TQ}}$. Hence *T*_1_ satisfies the following equation:

$$\begin{array}{c} \begin{array}{cc} \frac{\partial T_{1}}{\partial t}+\nabla \cdot \left(uT_{1}\right)-\delta_{T}\nabla^{2}T_{1}= & \left(\underset{\text{activation}\;\text{by}\;\text{IL}-12}{\underset{︸}{{\hat{\lambda }}_{T_{1}I_{12}}T_{10}\frac{I_{12}}{K_{I_{12}}+I_{12}}\frac{D}{K_{D}+D}}}\;+\underset{\text{promotion}\;\text{by}\;\text{IL}-2}{\underset{︸}{\lambda_{T_{1}I_{2}}T_{1}\frac{I_{2}}{K_{I_{2}}+I_{2}}}}\right)\;\\ & \times \underset{\text{inhibition}\;\text{by}\;\text{PD}-1-\text{PD}-\text{L}1}{\underset{︸}{\frac{1}{1+Q/K_{TQ}}}}\;-\underset{\text{death}}{\underset{︸}{d_{T_{1}}T_{1}}}. \end{array} \end{array}$$ (8)

**Equation for CD8^+^ T cells (*T*_8_)**. IL-12 activates CD8^+^ T cells and IL-2 induces proliferation of CD8^+^ T cells [44, 45]. Hence, similarly to the equation for *T*_1_, *T*_8_ satisfies the following equation:

$$\begin{array}{c} \begin{array}{cc} \frac{\partial T_{8}}{\partial t}+\nabla \cdot \left(uT_{8}\right)-\delta_{T}\nabla^{2}T_{8}= & \left(\underset{\text{activation}\;\text{by}\;\text{IL}-12}{\underset{︸}{{\hat{\lambda }}_{T_{8}I_{12}}T_{80}\frac{I_{12}}{K_{I_{12}}+I_{12}}\frac{D}{K_{D}+D}}}\;+\underset{\text{promotion}\;\text{by}\;\text{IL}-2}{\underset{︸}{\lambda_{T_{8}I_{2}}T_{1}\frac{I_{2}}{K_{I_{2}}+I_{2}}}}\;\right)\\ & \times \underset{\text{inhibition}\;\text{by}\;\text{PD}-1-\text{PD}-\text{L}1}{\underset{︸}{\frac{1}{1+Q/K_{TQ}}}}\;-\underset{\text{death}}{\underset{︸}{d_{T_{8}}T_{8}}}. \end{array} \end{array}$$ (9)

**Equation for IL-12 (*I*_12_)**. IL-12 is produced by activated DCs, so that

$$\begin{array}{ccc} \frac{\partial I_{12}}{\partial t}-\delta_{I_{12}}\nabla^{2}I_{12} & = & \underset{\text{production}\;\text{by}\;\text{DCs}}{\underset{︸}{\lambda_{I_{12}D}D}}\;-\underset{\text{degradation}}{\underset{︸}{d_{I_{12}}I_{12}}}. \end{array}$$ (10)

The diffusion coefficient of *I*_12_ is several orders of magnitude larger than the diffusion coefficient of cells. Hence the transport term ∇ ⋅ (**u***I*_12_) is negligible compared to the diffusion term $\delta_{I_{12}}\nabla^{2}I_{12}$, and it was therefore omitted.

**Equation for IL-2 (*I*_2_)**. IL-2 is produced by activated CD4^+^ T cells. Hence,

$$\begin{array}{ccc} \frac{\partial I_{2}}{\partial t}-\delta_{I_{2}}\nabla^{2}I_{2} & = & \underset{\text{production}\;\text{by}\;{\text{T}}_{1}}{\underset{︸}{\lambda_{I_{2}T_{1}}T_{1}}}\;-\underset{\text{degradation}}{\underset{︸}{d_{I_{2}}I_{2}}}. \end{array}$$ (11)

Here again the transport term was omitted.

**Equation for PD-1 (*P*), PD-L1 (*L*) and PD-1-PD-L1 (*Q*)**. PD-1 is expressed on the surface of activated CD4^+^ T cells and activated CD8^+^ T cells. Hence, *P* is given by *P* = *ρ*_*P*_(*T*_1_ + *T*_8_), where *ρ*_*P*_ is the ratio of the mass of all the PD-1 proteins in one T cell to the mass of one T cell. Thus, *P* satisfies the equation

$$\begin{array}{c} \begin{array}{cc} \frac{\partial P}{\partial t}+\nabla \cdot \left(uP\right)-\delta_{T}\nabla^{2}P & =\rho_{P}[\frac{\partial (T_{1}+T_{8})}{\partial t}+\nabla \cdot \left(u\left(T_{1}+T_{8}\right)\right)-\delta_{T}\nabla^{2}\left(T_{1}+T_{8}\right)], \end{array} \end{array}$$

or, by Eqs (8) and (9),

$$\begin{array}{c} \begin{array}{cc} \frac{\partial P}{\partial t}+\nabla \cdot \left(uP\right)-\delta_{T}\nabla^{2}P= & \rho_{P}[\left(\lambda_{T_{1}I_{12}}T_{10}+\lambda_{T_{8}I_{12}}T_{80}\right)\frac{I_{12}}{K_{I_{12}}+I_{12}}\\ & \;+\left(\lambda_{T_{1}I_{2}}T_{1}+\lambda_{T_{8}I_{2}}T_{8}\right)\frac{I_{2}}{K_{I_{2}}+I_{2}}]\times \frac{1}{1+Q/K_{TQ}}\\ & -\rho_{P}\left(d_{T_{1}}T_{1}+d_{T_{8}}T_{8}\right), \end{array} \end{array}$$

where $\rho_{P}=\frac{P}{T_{1}+T_{8}}$. Note that *P* undergoes the same advection velocity $\vec{u}$ as the T cells. We assume that PD-1 is depleted (or blocked) by *A* at rate *μ*_*PA*_*PA*, so that

$$\begin{array}{c} \begin{array}{cc} \frac{\partial P}{\partial t}+\nabla \cdot \left(uP_{1}\right)-\delta_{T}\nabla^{2}P= & \frac{P}{T_{1}+T_{8}}[\left(\lambda_{T_{1}I_{12}}T_{10}+\lambda_{T_{8}I_{12}}T_{80}\right)\frac{I_{12}}{K_{I_{12}}+I_{12}}\\ & +\left(\lambda_{T_{1}I_{2}}T_{1}+\lambda_{T_{8}I_{2}}T_{8}\right)\frac{I_{2}}{K_{I_{2}}+I_{2}}]\times \frac{1}{1+Q/K_{TQ}}\\ & -\frac{P}{T_{1}+T_{8}}\left(d_{T_{1}}T_{1}+d_{T_{8}}T_{8}\right)-\underset{\text{depletion}\;\text{by}\;\text{anti}-\text{PD}-1}{\underset{︸}{\mu_{PA}PA.}}\; \end{array} \end{array}$$ (12)

In the sequal we take the dimension of *μ*_*PA*_ to be cm^3^/g ⋅ day so that *A* is given in unit of g/cm^3^.

PD-L1 is expressed on the surface of activated CD4^+^ T cells, activated CD8^+^ T cells, and on tumor cells. Hence, the concentration of PD-L1 (*L*) is proportional to (*T*_1_ + *T*_8_) and *C*:

$$\begin{array}{c} L=\rho_{L}\left(T_{1}+T_{8}+\varepsilon C\right), \end{array}$$ (13)

where *ρ*_*L*_ is the ratio of the mass of all the PD-L1 proteins in one T cell to the mass of one T cell, and *ε* depends on the specific type of tumor.

PD-1 and PD-L1 form a complex PD-1-PD-L1 (*Q*), with association and disassociation rates *α*_*PL*_ and *d*_*Q*_, respectively:

$$\begin{array}{c} P+L\overset{\alpha_{PL}}{\underset{d_{Q}}{⇌}}Q. \end{array}$$ (14)

The half-life of *Q* is less then 1 second (i.e. 1.16 × 10^−5^ day) [47]. Hence, we may assume that the dynamics in (14) is in quasi-steady state, so that *α*_*PL*_ *PL* = *d*_*Q*_ *Q*, or

$$\begin{array}{c} Q=\sigma PL, \end{array}$$ (15)

where *σ* = *α*_*PL*_/*d*_*Q*_.

**Equation for anti-PD-1 (*A*)**. We assume that anti-PD-1 is injected intraperitoneally in the amount *γ*_*A*_ at the same days *t*_1_, *t*_2_, …, *t*_*n*_ as in the injection of virus. The PK/PD effect of the drug is assumed to be $\sum_{j=1}^{n}\gamma_{A}H(t-t_{j})e^{-\alpha (t-t_{j})}$, where *H*(*s*) = 0 if *s* ≤ 0, *H*(*t*) = 1 if *s* > 1. The drug *A* is depleted in the process of blocking PD-1. Hence,

$$\begin{array}{c} \begin{array}{cc} \frac{\partial A}{\partial t}-\delta_{A}\nabla^{2}A= & \underset{\text{injection}}{\underset{︸}{\sum_{j=1}^{n}\gamma_{A}H\left(t-t_{j}\right)e^{-\alpha \left(t-t_{j}\right)}}}-\underset{\text{depletion}\;\text{through}\;\text{blocking}\;\text{PD-1}}{\underset{︸}{\mu_{AP}PA}}\;\\ & -\underset{\text{degradation}}{\underset{︸}{d_{A}A}}. \end{array} \end{array}$$ (16)

**Equation for cells velocity (**u**)**: Cells disperse within the tissue, and its random motility may vary from one cell type to another. If the differences in the dispersion coefficients are ignored, then by adding the equations for all the cells and using Eq (1), we get

θ×∇·u=Right-handsideofEqs(2),(3)and(6)–(9).

To simplify the model we assume that the differences between the dispersion coefficients of the different cell types are small (but see comments in “Parameter estimation” (in “Diffusion coefficients”) and “Sensitivity analysis”), and proceed to use the above equation for ∇ ⋅ **u**.

We assume that the average density of each cell type eventually stabilizes with the following values: For cancer cells, 0.4 g/cm^3^; for dendritic cells 0.4 × 10^−4^ g/cm^3^; for macrophage, 0.2 g/cm^3^; for *T*_1_ cells, 2 × 10^−3^ g/cm^3^; and for *T*_8_ cells, 1 × 10^−3^ g/cm^3^. Recalling Eq (1) we find that *θ* = 0.6034, so that

$$\begin{array}{c} 0.6034\times \nabla \cdot u=\text{RHS}\;\text{of}\;\text{Eq}\;(2)+\text{RHS}\;\text{of}\;\text{Eq}\;(3)+\sum_{j=6}^{9}[\text{RHS}\;\text{of}\;\text{Eq}\;(\text{j})]. \end{array}$$ (17)

To simplify the computations, we assume that the tumor is spherical with moving boundary *r* = *R*(*t*), and that all the densities and concentrations are radially symmetric, that is, functions of (*r*, *t*), where 0 ≤ *r* ≤ *R*(*t*). In particular, **u** = *u*(*r*, *t*)**e**_*r*_, where **e**_*r*_ is the unit radial vector.

**Equation for free boundary (*R*)**: We assume that the free boundary *r* = *R*(*t*) moves with the velocity of cells, so that

$$\begin{array}{c} \frac{dR(t)}{dt}=u\left(R\left(t\right),t\right). \end{array}$$ (18)

**Boundary conditions** We assume that naive CD4^+^ T cells and CD8^+^ T cells which migrated from the lymph nodes into the tumor microenvironment have constant densities ${\hat{T}}_{8}$ at the tumor boundary, and that they are activated by dentritic cells and IL-12 upon entering the tumor. We represent this process by the flux conditions at the boundary:

$$\begin{array}{c} \frac{\partial T_{8}}{\partial n}+\sigma_{T}\left(I_{12}\right)\left(T_{8}-{\hat{T}}_{8}\right)=0,\;\frac{\partial T_{1}}{\partial n}+\sigma_{T}\left(I_{12}\right)\left(T_{1}-{\hat{T}}_{1}\right)=0\;\text{at}\;\;r=R\left(t\right), \end{array}$$ (19)

where $\sigma_{T}(I_{12})=\alpha_{T}\frac{I_{12}}{K_{I_{12}+I_{12}}}\frac{D}{K_{D}+D}$.

We impose zero-flux boundary condition on all the remaining variables:

$$\begin{array}{c} \begin{array}{c} \text{zero}-\text{flux}\;\text{for}\;C,\;C_{i},\;V_{e},\;V_{i},\;M,\;D,\;I_{12},\;I_{2},\;P,\;A\left(r,t\right)\;\text{at}\;\;r=R\left(t\right). \end{array} \end{array}$$ (20)

It is implicity assumed that receptors *P* become expressed only after *T*_1_ and *T*_8_ cells were already inside the tumor.

**Initial conditions** We prescribe the following values (in unit g/cm^3^) at day *t* = 0:

$$\begin{array}{c} \begin{array}{cc} & R(0)=0.01\;\text{cm},\;\text{and}\;\;C=0.3583,\\ & C_{i}=0,\;V_{e}=0,\;V_{i}=0,\;T_{1}=3\times 10^{-3},\;T_{8}=1.5\times 10^{-3},\;M=0.24,\\ & D=6\times 10^{-4},I_{2}=3.5\times 10^{-11},I_{12}=12\times 10^{-10},\;P=1.2\times 10^{-9}\;g/{\text{cm}}^{3}, \end{array} \end{array}$$ (21)

Note that the initial values satisfy Eq (1). We took the initial values for cells to be different from their above assumed asymptotic values. The choice of the initial conditions have little effect on the simulation results after a few days.

## Results

The simulations of the model were performed by Matlab based on the moving mesh method for solving partial differential equations with free boundary [48] (see the section on computational method).

**Fig 2:**
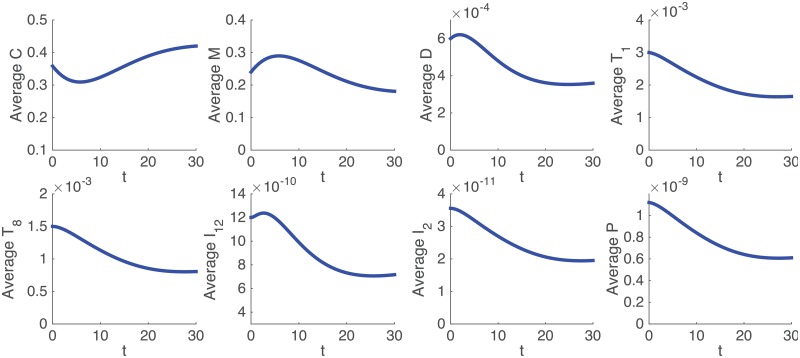
Average densities/concentrations, in g/cm^3^, of all the variables in the model in the control case. All parameter values are the same as in Tables 2 and 3. Initial values are as in (21).

Fig 2 shows the profiles of the average densities/concentrations of all the variables of the model in the first 30 days in the control case, that is, without treatment. The simulation results show that the steady states of all the cytokines and cells are approximately equal to the half-saturation values that we assumed in estimating the parameters of the model.

We proceed to simulate the treatment of cancer by OV and anti-PD-1 as single agents, and by a combination of the two drugs. Following mice experiments reported in [49], we apply the OV injections in days 0,2,4, and anti-PD-1 injection in days 4,7,11. From Figs 1(b) and 2(b) in [49] we see that although all the mice were identical and were treated with the same amounts of dose, their responses were varied: For some mice the tumor volume grew faster with OV than with anti-PD-1 as single agents, while for others this was the reverse, which means that the “effective” dose amounts varied with each subject. We account for this, in our model, by taking for each mouse somewhat different values of *γ*_*V*_ and *γ*_*A*_ which represent the effective doses for this subject. We also note that *γ*_*V*_ and *γ*_*A*_ should be approximately proportional to the amount of dose injected in the experiments. We determined the proportionality coefficients, or rather the orders of the magnitude of *γ*_*V*_ and *γ*_*A*_, so that the doubling time of the tumor volume (under treatment with a single agent) is approximately 20 days, which is the case for a large number of the mice in Figs 1(b), 2(b) of [49].

**Fig 3:**
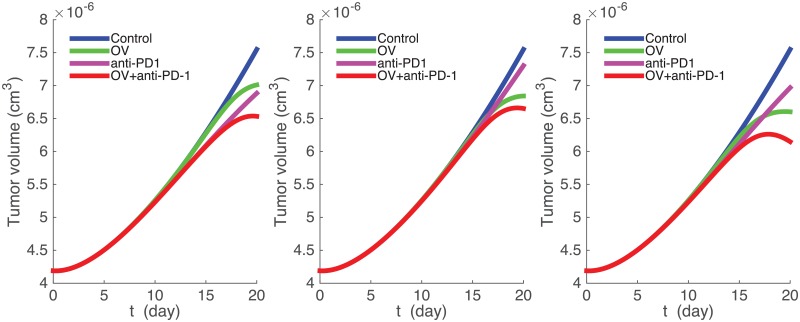
The growth of tumor volume. OV is given at days *t* = 0, 2, 4 with the amount *γ*_*V*_ and anti-PDE-1 is given at days *t* = 4, 7, 11 with the amount *γ*_*A*_. (a) *γ*_*V*_ = 0.1 × 10^−10^ g/cm^3^, *γ*_*A*_ = 8 × 10^−7^ g/cm^3^. (b) *γ*_*V*_ = 0.2 × 10^−10^ g/cm^3^, *γ*_*A*_ = 3 × 10^−7^ g/cm^3^. (c) *γ*_*V*_ = 0.5 × 10^−10^ g/cm^3^, *γ*_*A*_ = 7 × 10^−7^ g/cm^3^. Parameter values are the same as in Fig 2.

Fig 3(a)–3(c) show some simulations of the model with different values of *γ*_*V*_ and *γ*_*A*_. The profiles are similar to many of those given in [49]. In Fig 3(a) treatment with anti-PD-1 as single agent reduces tumor growth more than treatment with OV as single agent, and in Fig 3(b) and 3(c), it is the reverse, in agreement with profiles in [49]. In all cases, the combination reduces the tumor growth more than a single agent.

We can characterize the anticancer effectiveness of a virus by (i) its ability to replicate within cancer cells, as represented by the parameters $\lambda_{V_{i}}$ in Eq (5), and (ii) by its ability to stimulate the anticancer immune response, as represented by the activation rate λ_*DV*_ in Eq (7). For any pair ($\lambda_{V_{i}}$, λ_*DV*_) we may associate a “virtual virus” having these two parameters.

We proceed to use the mathematical model to conduct *in silico* clinical trials. As an example, a treatment will be given for a period of 16 weeks, and the patient’s tumor volume will be measured at the end of 24 weeks from the initial treatment. The virus is injected into the tumor at the beginning of weeks 1,3,5,7,9,11,13 and 15, at an amount *γ*_*V*_, and the anti-PD-1 is given at the beginning of weeks 1,4,7,10, 13, 16 at an amount *γ*_*A*_. We denote by *V*_24_(*γ*_*V*_, *γ*_*A*_) the volume of the tumor at the end of 24 weeks, and define the efficacy of the treatment by the formula:

$$\begin{array}{c} E\left(\gamma_{V},\gamma_{A}\right)=\frac{V_{24}\left(0,0\right)-V_{24}\left(\gamma_{V},\gamma_{A}\right)}{V_{24}\left(0,0\right)}; \end{array}$$

thus the efficacy is increased if the tumor volume *V*_24_(*γ*_*V*_, *γ*_*A*_) is decreased.

**Fig 4:**
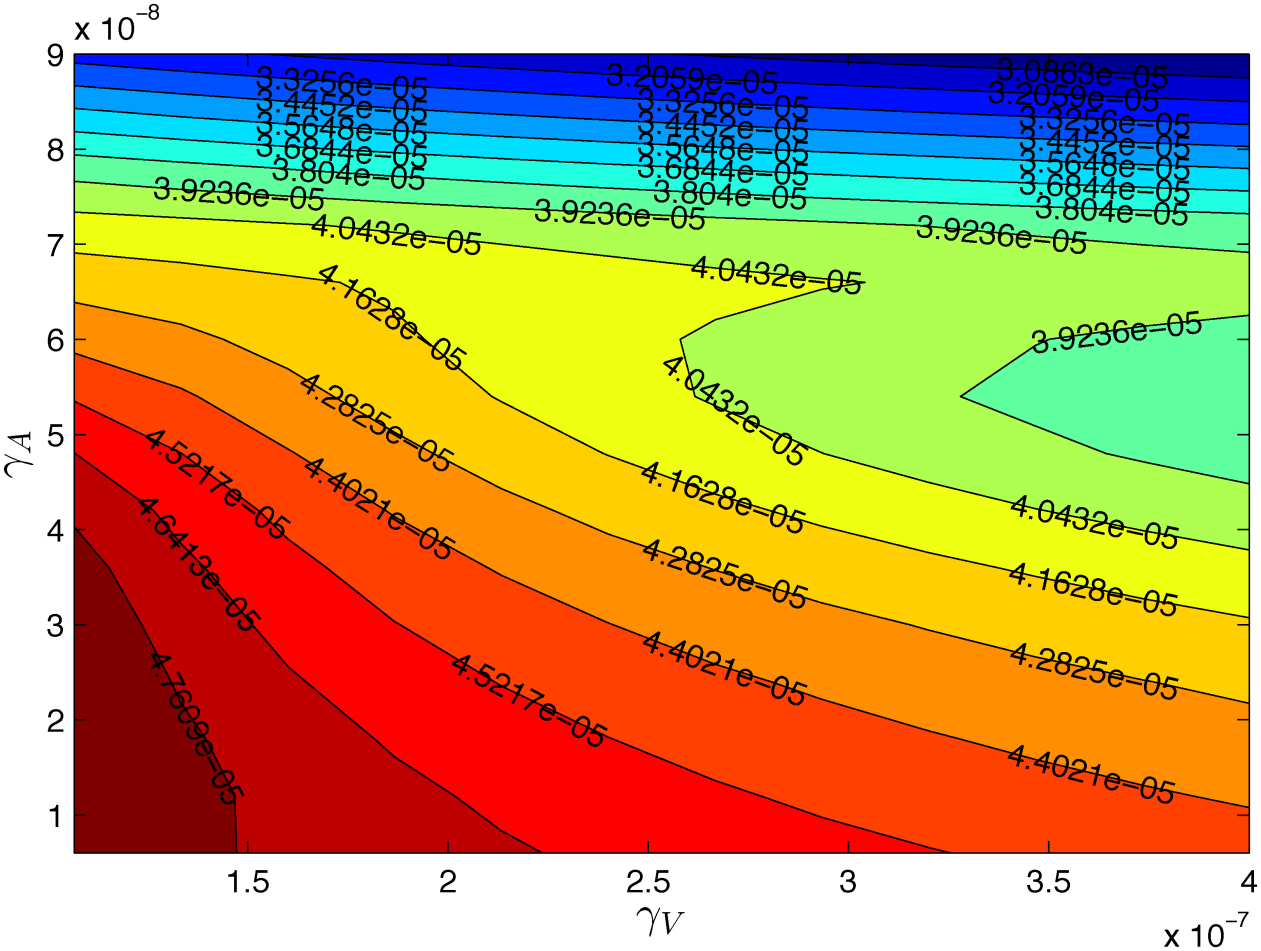
The tumor volume at week 24 for different pair of (*γ*_*V*_, *γ*_*A*_). Here $\lambda_{V_{i}}=5\times 10^{-4}/\text{day}$, λ_*DV*_ = 5.2 × 10^10^ cm^3^/g ⋅ day, *γ*_*V*_ = 1 × 10^−7^−4 × 10^−7^ g/cm^3^ and *γ*_*A*_ = 0.6 × 10^−8^−9 × 10^−8^ g/cm^3^. All other parameter values are the same as in Tables 2 and 3.

**Fig 5:**
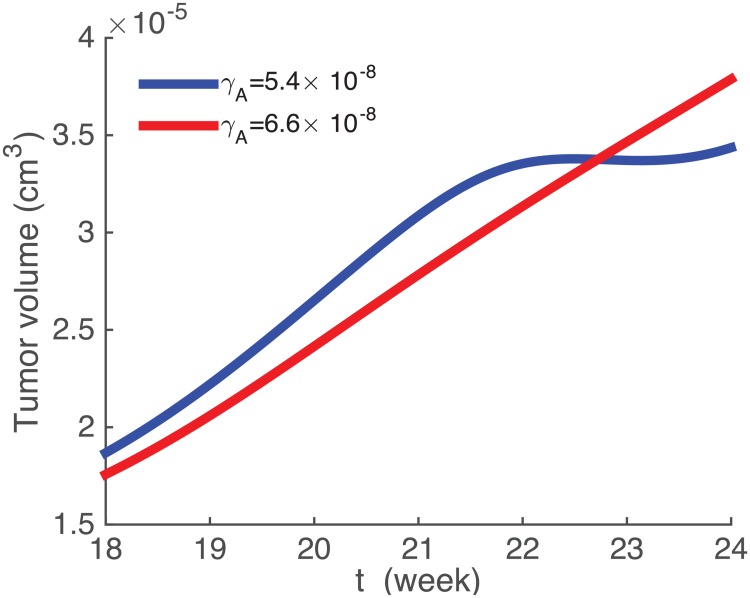
Growth of tumor volume. Here, *γ*_*V*_ = 3.7 × 10^−7^ g/cm^3^. Other parameter values are the same as in Fig 4.

**Fig 6:**
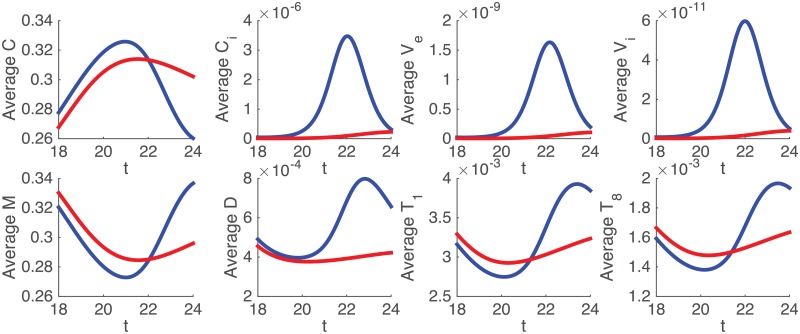
The average densities and tumor volume. Blue: *γ*_*V*_ = 3.7 × 10^−7^ g/cm^3^, *γ*_*A*_ = 5.4 × 10^−8^ g/cm^3^. Red: *γ*_*V*_ = 3.7 × 10^−7^ g/cm^3^, *γ*_*A*_ = 6.6 × 10^−8^ g/cm^3^. Other parameter values are the same as in Fig 4.

Fig 4 shows an efficacy map for the parameters $\lambda_{V_{i}}=5\times 10^{-4}/\text{day}$, λ_*DV*_ = 5.2 × 10^10^ cm^3^/g ⋅ day. For clarity we marked tumor volumes *V*_24_(*γ*_*V*_, *γ*_*A*_) on the equi-efficacy curves. We see that as *γ*_*V*_ increases so does the efficacy. However, the same is not true of *γ*_*A*_: there are regions where the efficacy decreases as *γ*_*A*_ increases. To understand what happens in such regions we take two points with the same *γ*_*V*_: (3.7 × 10^−7^, 5.4 × 10^−8^) and (3.7 × 10^−7^, 6.6 × 10^−8^). Fig 5 shows that the tumor volume for the larger *γ*_*A*_ is somewhat larger than the tumor volume for the smaller *γ*_*A*_. Fig 6 explains what has actually occurred. With the higher dose, more infected cancer cells were killed, and the virus population decreased. Hence the number of activated dendritic cells decreased and then also the number of T cells decreased, which resulted in an increase in the number of uninfected cancer cells.

**Fig 7:**
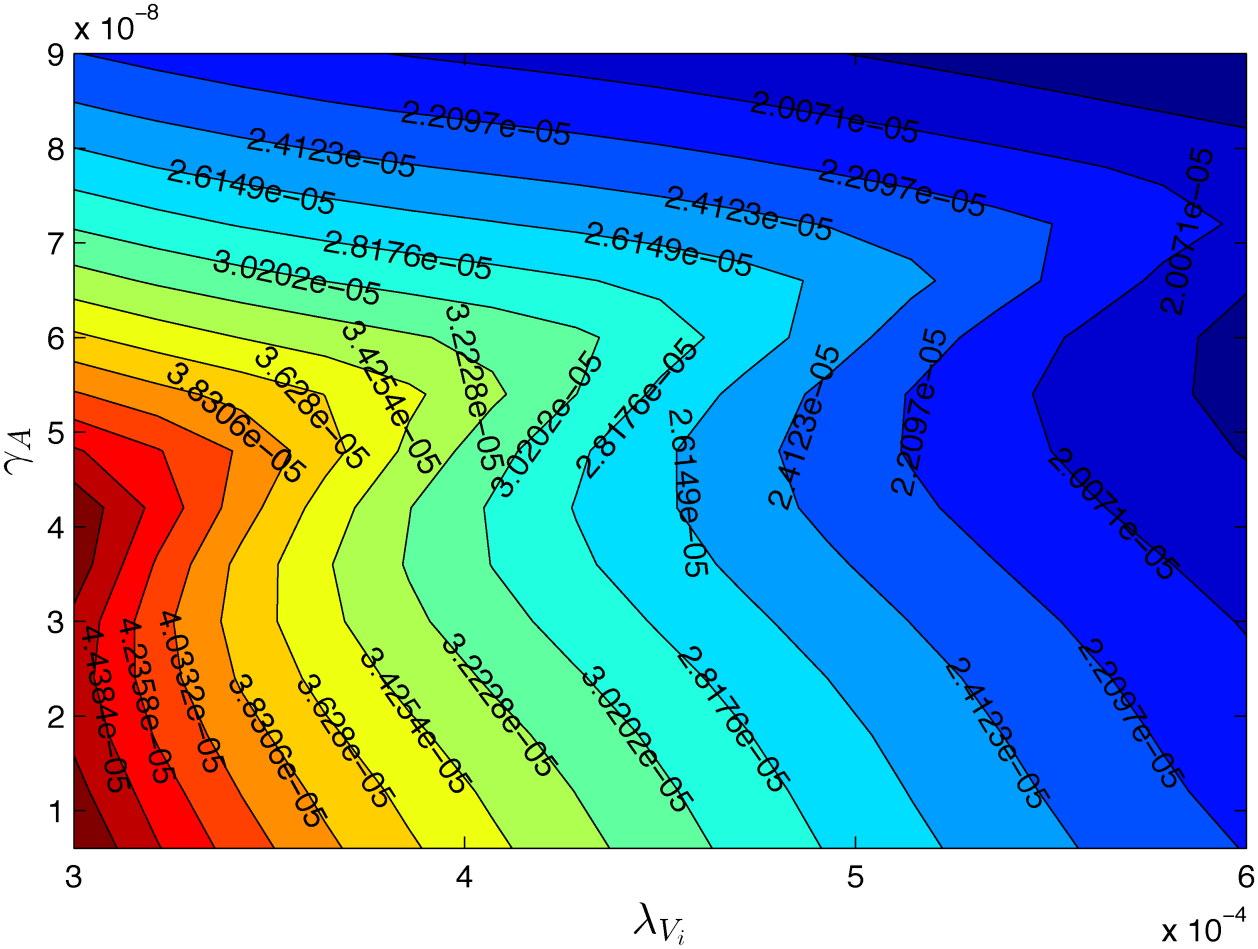
The tumor volume at week 24. Here, *γ*_*V*_ = 2.5 × 10^−7^ g/cm^3^ and λ_*DV*_ = 5.2 × 10^10^ cm^3^/g⋅day. All other parameter values are the same as in Tables 2 and 3.

We find the same phenomenon in Fig 7, which is an efficacy map for (*γ*_*A*_, $\lambda_{V_{i}}$), for specific values of *γ*_*V*_ = 2.5 × 10^−7^ g/cm^3^/day and λ_*DV*_ = 5.2 × 10^10^ cm^3^/g ⋅ day. The tumor volume decreases as $\lambda_{V_{i}}$ increases, but there are values of $\lambda_{V_{i}}$ for which the tumor volume increases when *γ*_*A*_ is increased.

We note that λ_*DV*_ and *γ*_*A*_ are positively correlated, since both are increasing the activity of effector T cells. We therefore expect that, unlike situation in Fig 4, the tumor volume will decrease as *γ*_*A*_ increases.

One is tempted to replace PDE system by a simpler system of ODEs where the diffusion and advection terms are dropped. However, since the diffusion of cells is several orders of magnitude smaller than diffusion of cytokines and extracellular virus, the ODE system cannot adequately represent the PDE model. For example, Fig 6 show antagonism between the OV and anti-PD-1, whereas this antagonism disappears in the ODE model.

## Conclusion

Oncolytic virus (OV) is a genetically modified virus that can selectively invade cancer cells and replicate inside them. When an infected cell dies, its virus particles are released and proceed to infect other cancer cells. OV therapy, as a single agent, had not been successful because macrophages recognize infected cells and kill them together with their viruses. Recent studies use new designs of OV that can stimulate cytotoxic T cells to kill cancer cells before the viral population is significantly depleted by the macrophages. Some of these studies introduce enhancement of the T cells by blocking their checkpoints. Mice experiments demonstrated that both CTLA-4 and PD-L1 checkpoints blockade enhance the OV treatment [29–33]. There are recent clinical trials with OV and checkpoint inhibitors [34–37]. In particular, in clinical trials for melanoma, reported in [36], patients were treated with OV (T-VEC) and anti-CTLA-4 (ipilimumab) for a period of 13 weeks and were observed for an average period of 20 months.

Since T cells kill not only virus-free cancer cells but also virus-infected cancer cells, they may disrupt the anti-cancer effect of the OV. Hence an increase in the dose of the checkpoint inhibitor may actually have a pro-cancer effect. In order to clarify this situation we developed a mathematical model that includes the immune cells (macrophages, dendritic cells, and effective T cells), and characterized the OV by two parameters: the replication potential ($\lambda_{V_{i}}$) and its immunogenicity potential (λ_*DV*_). We first simulated a treatment corresponding to mice experiments, where the dose *γ*_*A*_ of anti-PD-1 and the dose *γ*_*V*_ of OV were administered for 11 days, and the tumor volume was observed for 30 days. We found quantitative agreement with experimental results [49].

We then proceeded to use the model to run in *silico* clinical trials, where the treatment with a combination (*γ*_*V*_, *γ*_*A*_) was given for 14 weeks, and we observed the results of the treatment for 24 weeks. We simulated the tumor volume *V*_24_(*γ*_*V*_, *γ*_*A*_) at the end of 24 weeks for a range of (*γ*_*V*_, *γ*_*A*_). We found that there are regions in the (*γ*_*V*_, *γ*_*A*_)-plane where an increase in *γ*_*A*_ results in an increase in the tumor volume. Thus, there are regions of antagonism between the two drugs, where an increase in the anti-PD-1 decreases the efficacy of the treatment. We also simulated *V*_24_ ($\lambda_{V_{i}}$, *γ*_*A*_) for a fixed parameter *γ*_*V*_ and variable ($\lambda_{V_{i}}$, *γ*_*A*_). We again found regions of antagonism where an increase in *γ*_*A*_ results in an increase in the tumor volume.

These results have implications for clinical trials. Indeed for a clinical trial to be successful, the regions of antagonism between the doses of the checkpoint inhibitor and the OV doses should be determined early on, and avoided; these regions may depend on the specific oncolytic virus which is used in the clinical trial.

## Materials and methods

### Parameter estimation

**Fig 8:**
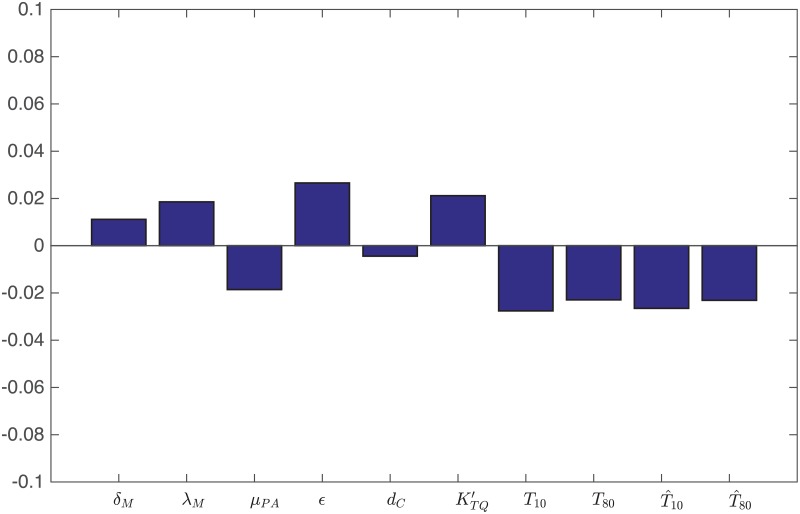
Statistically significant PRCC values (p-value< 0.01) for *R*(*t*) at day 60.

**Fig 9:**
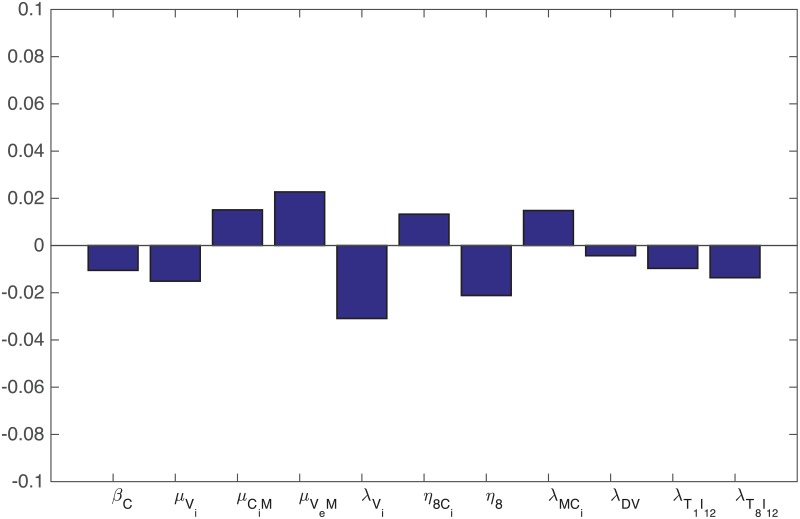
Statistically significant PRCC values (p-value< 0.01) for *R*(*t*) at day 60.

Many of parameters in Tables 2 and 3 were taken directly from [38, 39, 50, 51]. When a value taken from these references was not obtained directly from experimental papers or was not carefully estimated from experimental results, we added an asterisk “*” next to the reference. These parameters were used in sensitivity analysis (see Figs 8 and 9) in order to see how the tumor value is affected by a random increase or decrease of these parameters by a factor of 2; but the dispersion coefficient of macrophages was increased by up to a factor of 4 because they are highly mobile.

**Table 2:** Summary of parameter values.

Notation	Description	Value used	References
*δ*_*D*_	diffusion coefficient of DCs	8.64 × 10^−7^ cm^2^ day^−1^	[38]*
$\delta_{T_{1}}$	diffusion coefficient of CD4^+^ T cells	8.64 × 10^−7^ cm^2^ day^−1^	[38]*
$\delta_{T_{8}}$	diffusion coefficient of CD8^+^ T cells	8.64 × 10^−7^ cm^2^ day^−1^	[38]*
*δ*_*C*_	diffusion coefficient of tumor cells	8.64 × 10^−7^ cm^2^ day^−1^	[38]*
*δ*_*M*_	diffusion coefficient of macrophages	8.64 × 10^−7^ cm^2^ day^−1^	[38]*
$\delta_{I_{12}}$	diffusion coefficient of IL-12	6.0472 × 10^−2^ cm^2^ day^−1^	[39]
$\delta_{I_{2}}$	diffusion coefficient of IL-2	9.9956 × 10^−2^ cm^2^ day^−1^	[39]
*δ*_*A*_	diffusion coefficient of IL-2	4.73 × 10^−2^ cm^2^ day^−1^	[39]
*α*_*T*_	flux rate of T cells on the boundary	1 cm^−1^	estimated
λ_*C*_	growth rate of cancer cells	0.65 day^−1^	estimated
$\lambda_{V_{i}}$	growth rate of intracellular virus	6 × 10^−4^ day^−1^	estimated
λ_*M*_	growth rate of macrophages	0.009 day^−1^	[51]*
$\lambda_{MC_{i}}$	activation rate of macrophages by *C*_*i*_	0.04 cm^3^/g	estimated
λ_*DV*_	activation rate of DCs by virus infection	5.2 × 10^10^ cm^3^/g ⋅ day	estimated
λ_*DC*_	activation rate of DCs by tumor cells	5.2 day^−1^	estimated
$\lambda_{T_{1}I_{12}}$	activation rate of CD4^+^ T cells by IL-12	9.32 day^−1^	[39]
$\lambda_{T_{1}I_{2}}$	activation rate of CD4^+^ T cells by IL-2	0.25 day^−1^	[39]
$\lambda_{T_{8}I_{12}}$	activation rate of CD8^+^ T cells by IL-12	8.30 day^−1^	[39]
$\lambda_{T_{8}I_{2}}$	activation rate of CD8^+^ T cells by IL-2	0.25 day^−1^	[39]
λ_*I*_12_*D*_	production rate of IL-12 by DCs	2.76 × 10^−6^ day^−1^	[39]
$\lambda_{I_{2}T_{1}}$	production rate of IL-2 by CD4^+^ T cells	2.82 × 10^−8^ day^−1^	[39]
*β*_*C*_	infection rate of cancer cells by virus	9 × 10^4^ cm^3^/g ⋅ day	estimated
*β*_*V*_	rate of transition from *V*_*e*_ to *V*_*i*_ by infection	0.09 cm^3^/g ⋅ day	estimated
*μ*_*C*_*i*_*M*_	killing rate of *C*_*i*_ by *M*	4.8 × 10^−2^ cm^3^/g ⋅ day	estimated
*μ*_*V*_*e*_*M*_	clearance rate of *V*_*e*_ by *M*	2 cm^3^/g ⋅ day	estimated
$\mu_{V_{i}}$	death rate of infected cell due to viral burden	5 × 10^7^ day^−1^	estimated
*N*	burst size of *V*_*i*_ from natural death of *C*_*i*_	100	estimated
*η*_8_	killing rate of tumor cells by CD8^+^ T cells	1.38 × 10^2^ day^−1^ ⋅ cm^3^/g	estimated
$\eta_{8C_{i}}$	killing rate of infected cancer cells by CD8^+^ T cells	7.59 × 10^3^ day^−1^ ⋅ cm^3^/g	estimated
*μ*_*PA*_	blocking rate of PD-1 by anti-PD-1	6.87 × 10^4^ cm^3^/g ⋅ day	[39]
*ρ*_*P*_	expression of PD-1 in T cells	2.49 × 10^−7^	[39]
*ρ*_*L*_	expression of PD-L1 in T cells	5.22 × 10^−7^	[39]
*ε*	relative expression of PD-L1 in tumor cells	0.01	[39]
*d*_*C*_	death rate of uninfected tumor cells	0.17 day^−1^	[50]^*^
*d*_*M*_	death rate of macrophages	0.015 day^−1^	[51]
*d*_*D*_	death rate of DCs	0.1 day^−1^	[50]
$d_{T_{1}}$	death rate of CD4^+^ T cells	0.197 day^−1^	[50]
$d_{T_{8}}$	death rate of CD8^+^ T cells	0.18 day^−1^	[50]
$d_{I_{12}}$	degradation rate of IL-12	1.38 day^−1^	[50]
$d_{I_{2}}$	degradation rate of IL-2	2.376 day^−1^	[50]

* In this reference the value was estimated but not obtained directly from experimental results.

**Table 3:** Summary of parameter values.

*K*_*C*_	half-saturation of tumor cells	0.4 g/cm^3^	[50]
*K*_*D*_	half-saturation of DCs	0.4 × 10^−4^ g/cm^3^	[39]
$K_{I_{12}}$	half-saturation of IL-12	1.5 × 10^−10^ g/cm^3^	[50]
$K_{I_{2}}$	half-saturation of IL-2	2.37 × 10^−11^ g/cm^3^	[50]
$K_{T_{1}}$	half-saturation of CD4^+^ T cells	2 × 10^−3^ g/cm^3^	[39]
$K_{T_{8}}$	half-saturation of CD8^+^ T cells	1 × 10^−3^ g/cm^3^	[39]
$K_{TQ}^{\prime }$	inhibition of function of T cells by PD-1-PD-L1	1.365 × 10^−18^ g/cm^3^	[39]*
*θ*	total cell density	0.6034 g/cm^3^	**
*D*_0_	density of immature DCs	2 × 10^−5^ g/cm^3^	[50]
*T*_10_	density of naive CD4^+^ T cells	4 × 10^−4^ g/cm^3^	[39]*
*T*_80_	density of naive CD8^+^ T cells	2 × 10^−4^ g/cm^3^	[39]*
*C*_*M*_	carrying capacity of cancer cells	0.8 g/cm^3^	[50]
${\hat{T}}_{1}$	density of CD4^+^ T cells from lymph node	4 × 10^−3^ g/cm^3^	[39]*
${\hat{T}}_{8}$	density of CD8^+^ T cells from lymph node	2 × 10^−3^ g/cm^3^	[39]*

* In this reference the value was estimated but not obtained directly from experimental results.

** The value is determined by Eq (1) with steady state densities of the cells.

#### Diffusion coefficients

The diffusion coefficients of cytokines were computed in [39] based on the formula

$$\begin{array}{c} \delta_{p}=\frac{A}{M_{p}^{1/3}}, \end{array}$$

where *A* is a constant and *p* is any protein with diffusion coefficient *δ*_*p*_ and molecular weight *M*_*p*_. The diffusion coefficients of cells may vary depending on the cell type. For simplicity we take them equal, and choose the common value as in [39], but we show in the section on sensitivity analysis that the tumor growth is affected very little by taking different diffusion coefficients for different cell types.

#### Half-saturation

In an expression of the form $Y\frac{X}{K_{X}+X}$ where *Y* is activated by *X*, the parameter *K*_*X*_ is called the *half-saturation* of *X*. If *X* reaches a steady state *X*_0_, we expect that *X*_0_/(*K*_*X*_ + *X*_0_) will not be “too close” to 0 and not “too close” to 1. For definiteness we take *X*_0_/(*K*_*X*_ + *X*_0_) to be 1/2, so that

$$\begin{array}{c} K_{X}=X_{0}; \end{array}$$

the steady state *X*_0_ is derived from experimental or clinical data.

#### Eq (2)

We take λ_*C*_ = 0.65/day, which is slightly smaller than in [38], and *β*_*C*_ = 9 × 10^4^ cm^3^/g ⋅ day, which is slightly larger than in [52], and we take *η*_8_ = 1.38 × 10^2^ cm^3^/g ⋅ day, which is slightly larger than in [39].

#### Eq (3)

We assume that *T*_8_ cells kill *C*_*i*_ much more efficiently than they kill *C*, and take $\eta_{8C_{i}}=55\eta_{8}=7.59\times 10^{3}\;{\text{cm}}^{3}/\text{g}\cdot \text{day}$. We assume that OV is designed to stimulate the immune system while it is in infected cells. Therefore the viral burden does not increase the death rate of infected cells very much. We therefore take $\mu_{V_{i}}=2\times 10^{7}\;{\text{cm}}^{3}/\text{g}$ so that $\mu_{V_{i}}$ *V_i_* is very small compared to 1, e.g. it increases the death of the infected cancer cell by 2% when the viral load is 10^−9^ g/cm^3^. Macrophages engulf infected cancer cells [40], but we assume that the rate is extremely small compared to the rate by which *T*_8_ kill infected cancer cells. We accordingly take *μ*_*C*_*i*_*M*_ = 4.8 × 10^−2^/day.

#### Eqs (4) and (5)

We take *N* = 100, which is in the range considered in [51]. We assume that the ratio of mass of one virus to one cell is *m*_*VC*_ = 10^−6^. Hence *β*_*V*_ = *β*_*C*_*m*_*VC*_ = 0.09 cm^3^/g ⋅ day. We assume that the clearance rate of *V*_*e*_ by macrophages is much larger than the rate by which *V*_*e*_ invades uninfected cancer cells, and take *μ*_*V*_*e*_*M*_ = 2 cm^3^/g ⋅ day. We assume that the ratio of *C*_*i*_/*V*_*i*_ in the first 12 days averages 3 × 10^6^, and that the replication of an intracellular virus occurs approximately every 22-23 hours, so that the growth rate per day is 1.5 × 2^9^*V*_*i*_. Hence $\lambda_{V_{i}}C_{i}=\lambda_{V_{i}}(\frac{C_{i}}{V_{i}})V_{i}=(\lambda_{V_{i}}\times 3\times 10^{6})V_{i}$ and the growth rate of *V*_*i*_ is then determined by the equation $(\lambda_{V_{i}}\times 3\times 10^{6})V_{i}=1.5\times 2^{9}V_{i}$, so that $\lambda_{V_{i}}=6\times 10^{-4}/day$.

#### Eq (6)

Without OV, we assume λ_*M*_ = *d*_*M*_ *M* in steady state, where *d*_*M*_ = 0.015/day and *M* = *K*_*M*_ = 0.2 g/cm^3^. Hence λ_*M*_ = 0.003/day. We note however that in estimating λ_*M*_, we ignored the contribution of ∇ ⋅ (**u***M*), whose integral over the tumor {*r* < *R*(*t*)} is $\int_{r=R(t)}\frac{dR(t)}{dt}\cdot M$, which is a positive quantity. Hence, $\frac{\partial M}{\partial t}$ is actually decreased when we equate to zero the right-hand side (RHS) of Eq (6); we therefore need to increase λ_*M*_; we take λ_*M*_ = 0.09/day. Since initially tumor is with radius *R*(0) = 0.01 cm, macrophages had already arrived into the tumor tissue so that the additional increase in macrophages, $\lambda_{MC_{i}}MC_{i}$, is assumed to be ‘relatively’ small; we take $\lambda_{MC_{i}}=0.04\;{\text{cm}}^{3}/\text{g}$.

#### Eq (7)

We take λ_*DC*_ = 5.2/day which is slightly larger than in [39]. We assume that the virus, although having decreased over time, is still effective in activating dendritic cells, so that λ_*DV*_ *V*_*i*_ is comparable to λ_*DC*_ when *V*_*i*_ ≈ 10^−10^ g/cm^3^. Accordingly, we take λ_*DV*_ = 5.2 × 10^10^ cm^3^/g ⋅ day.

### Sensitivity analysis

We performed sensitivity analysis with respect to the tumor volume at day 30 for two sets of parameters. The first set consists of parameters marked by “*” in Tables 2 and 3. These parameters were not derived, or not carefully estimated, from experimental or clinical data. These parameters are: *δ*_*C*_, *δ*_*M*_, *δ*_*D*_, $\delta_{T_{1}}$, $\delta_{T_{8}}$, λ_*M*_, *μ*_*PA*_, *ε*, *d*_*C*_, $K_{TQ}^{\prime }$, *T*_10_, *T*_80_, ${\hat{T}}_{1}$, ${\hat{T}}_{8}$. Following the method of [53], we performed Latin hypercube sampling and generated 5000 samples to calculate the partial rank correlation coefficients (PRCC) and the p-values with respect to the tumor volume at day 30. In sampling all the parameters, we took the range of each parameter (except the diffusion coefficients) from 1/2 to twice its value in Tables 2 or 3. In the simulations of the model we assumed that the diffusion coefficients of all the cell types are equal. What may cause a significant difference in the simulations is actually the differences between the diffusion coefficients of cell types, rather than their actual values. Since macrophages are highly mobile, we chose to include only *δ*_*M*_ in the sensitivity analysis, keeping all other diffusion coefficient equal, and randomly increasing *δ*_*M*_ by up to a factor of 4. The results are shown in Fig 8. We see that increasing the source of T cells ($T_{10},T_{80},{\hat{T}}_{10},{\hat{T}}_{80}$) decreases the tumor volume, as does the depletion rate (*μ*_*PA*_) of PD-1 by the PD-1 inhibitor. On the other hand the production rate of PD-L1 by the cancer (*ε*) increases the tumor volume. An increase of the random mobility of macrophages, by a factor up to 4, only slightly increases the tumor volume.

The second set of parameters in the sensitivity analysis are some production parameters, namely $\lambda_{V_{i}}$, $\lambda_{MC_{i}}$, λ_*DV*_, $\lambda_{T_{1}I_{12}}$ and $\lambda_{T_{8}I_{12}}$, and the parameters *β*_*C*_, $\mu_{V_{i}}$, *μ*_*C*_*i*_*M*_, *η*_8_, $\eta_{8C_{i}}$ and $d_{V_{e}M}$ which play important roles in the dynamics of *C*. Here again we sampled all the parameters by taking the range of each parameter for 1/2 to twice its value in Tables 2 and 3. The results are shown in Fig 9.

It is interesting to see from Fig 9 that the parameters that promote killing of infected cancer cells, such as *μ*_*C*_*i*_*M*_, *μ*_*V*_*e*_*M*_, $\eta_{8C_{i}}$ and $\lambda_{MC_{i}}$ are positively correlated with the tumor volume, while the parameters that promote viral infection, such as *β*_*C*_, $\mu_{V_{i}}$ and $\lambda_{V_{i}}$, are negatively correlated with the tumor volume. We also see that the production/activation rates that promote effector T cells, namely, λ_*DV*_, $\lambda_{T_{1}I_{12}}$ and $\lambda_{T_{8}I_{12}}$, are negatively correlated to the tumor volume, while the killing rate of uninfected cancer cells cells by CD8^+^ T cells, *η*_8_, is negatively correlated with tumor volume.

### Computational method

We employ moving mesh method [48] to numerically solve the free boundary problem for the tumor proliferation model. To illustrate this method, we take Eq (2) as example and rewrite it as the following form:

$$\begin{array}{c} \begin{array}{c} \frac{\partial C(r,t)}{\partial t}=\delta_{C}\Delta C\left(r,t\right)-div\left(uC\right)+F,\; \end{array} \end{array}$$ (22)

where *F* represents the term in the right hand side of Eq (2). Let $r_{i}^{k}$ and $C_{i}^{k}$ denote numerical approximations of i-th grid point and $C(r_{i}^{k},n\tau )$, respectively, where *τ* is the size of time-step. The discretization of Eq (22) is derived by the fully implicit finite difference scheme:

$$\begin{array}{c} \begin{array}{c} \frac{C_{i}^{k+1}-C_{i}^{k}}{\tau }=\delta_{C}(C_{rr}+\frac{2}{r_{i}^{k}}C_{r})-(\frac{2}{r_{i}^{k+1}}u_{i}^{k+1}+u_{r})C_{i}^{k+1}-u_{i}^{k+1}C_{r}+F_{i}^{k+1}, \end{array} \end{array}$$ (23)

where $C_{r}=\frac{h_{-1}^{2}C_{i+1}^{k+1}-h_{1}^{2}C_{i-1}^{k+1}-(h_{1}^{2}-h_{-1}^{2})C_{i}^{k+1}}{h_{1}(h_{-1}^{2}-h_{1}h_{-1})}$, $C_{rr}=2\frac{h_{-1}C_{i+1}^{k+1}-h_{1}C_{i-1}^{k+1}+(h_{1}-h_{-1})C_{i}^{k+1}}{h_{1}(h_{1}h_{-1}-h_{-1}^{2})}$, $u_{r}=\frac{h_{-1}^{2}u_{i+1}^{k+1}-h_{1}^{2}u_{i-1}^{k+1}-(h_{1}^{2}-h_{-1}^{2})u_{i}^{k+1}}{h_{1}(h_{-1}^{2}-h_{1}h_{-1})}$, $h_{-1}=r_{i-1}^{k+1}-r_{i}^{k+1}$ and $h_{1}=r_{i+1}^{k+1}-r_{i}^{k+1}$. The mesh moves by $r_{i}^{k+1}=r_{i}^{k}+u_{i}^{k+1}\tau$, where $u_{i}^{k+1}$ is solved by the velocity equation.
